# Influences of Parental Snacking-Related Attitudes, Behaviours and Nutritional Knowledge on Young Children’s Healthy and Unhealthy Snacking: The ToyBox Study

**DOI:** 10.3390/nu12020432

**Published:** 2020-02-07

**Authors:** Edward Leigh Gibson, Odysseas Androutsos, Luis Moreno, Paloma Flores-Barrantes, Piotr Socha, Violeta Iotova, Greet Cardon, Ilse De Bourdeaudhuij, Berthold Koletzko, Simona Skripkauskaite, Yannis Manios, on behalf of the Toybox-study Group

**Affiliations:** 1 Department of Psychology, University of Roehampton, London SW15 4JD, UK; s.skripkauskaite@bangor.ac.uk; 2 Department of Nutrition and Dietetics, School of Physical Education, Sport Science and Dietetics, University of Thessaly, 42132 Trikala, Greece; 3 GENUD (Growth, Exercise, NUtrition and Development) Research Group, Faculty of Health Sciences, University of Zaragoza, Edificio del SAI, C/Pedro Cerbuna s/n, 50009 Saragossa, Spain; lmoreno@unizar.es (L.M.); pfloresbarrantes@gmail.com (P.F.-B.); 4 Instituto Agroalimentario de Aragón (IA2), 50013 Saragossa, Spain; 5 Fundación Instituto de Investigación Sanitaria Aragón (IIS Aragón), 50009 Saragossa, Spain; 6 The Children’s Memorial Health Institute, 04-730 Warsaw, Poland; p.socha@ipczd.pl; 7 Department of Pediatrics, Medical University of Varna, 9002 Varna, Bulgaria; violeta.iotova@mu-varna.bg; 8 Department of Movement and Sports Sciences, Ghent University, 9000 Ghent, Belgium; greet.cardon@ugent.be (G.C.); Ilse.Debourdeaudhuij@UGent.be (I.D.B.); 9 Dr von Hauner Children’s Hospital, LMU-Ludwig-Maximilians-University at Munich, D-80337 Munich, Germany; Berthold.Koletzko@med.uni-muenchen.de; 10 Department of Nutrition and Dietetics, School of Health Science and Education, Harokopio University, 17671 Athens, Greece; manios.toybox@hua.gr

**Keywords:** child obesity, snacking, preschool children, nutrition, parents, feeding practices, Europe

## Abstract

This study investigated parental influences on preschool children’s healthy and unhealthy snacking in relation to child obesity in a large cross-sectional multinational sample. Parents and 3–5 year-old child dyads (*n* = 5185) in a kindergarten-based study provided extensive sociodemographic, dietary practice and food intake data. Parental feeding practices that were derived from questionnaires were examined for associations with child healthy and unhealthy snacking in adjusted multilevel models, including child estimated energy expenditure, parental education, and nutritional knowledge. Parental healthy and unhealthy snacking was respectively associated with their children’s snacking (both *p* < 0.0001). Making healthy snacks available to their children was specifically associated with greater child healthy snack intake (*p* < 0.0001). Conversely, practices that were related to unhealthy snacking, i.e., being permissive about unhealthy snacking and acceding to child demands for unhealthy snacks, were associated with greater consumption of unhealthy snacks by children, but also less intake of healthy snacks (all *p* < 0.0001). Parents having more education and greater nutritional knowledge of snack food recommendations had children who ate more healthy snacks (all *p* < 0.0001) and fewer unhealthy snacks (*p* = 0.002, *p* < 0.0001, respectively). In the adjusted models, child obesity was not related to healthy or unhealthy snack intake in these young children. The findings support interventions that address parental practices and distinguish between healthy and unhealthy snacking to influence young children’s dietary patterns.

## 1. Introduction

Trends in childhood obesity are a concern in many countries, with evidence for increasing prevalence of obesity, even in preschool children [1]. Numerous interventions have been attempted, addressing eating, drinking, and activity energy-balance related behaviours, primarily in school-aged children, but with mixed success [2,3,4]. In younger children, family environment, and particularly parental feeding practices, are strong predictors of both children’s diet [5] and adiposity [6], and so successful interventions should include these aspects.

Snacking is an important energy-balance related dietary behaviour in children: young children have proportionately the highest nutritional and energetic requirements of any stage of the lifespan [7]; thus, frequent eating, including snacking between meals, is typical, easily reinforced, and avoids acute nutritional deficits that might otherwise limit physical and psychological development [8,9]. Therefore, snacking can substantially contribute to daily energy intake; for example, one recent comparison of four national nutritional surveys of 4–13-year-old children found that snacking provided up to one-third of energy intake [10]. Furthermore, there is concern that children’s choice of snacks are often particularly high in sugar and fat [11,12]. Therefore, it is important to distinguish between snacking on foods with a healthy nutrient profile (e.g., fruit and vegetables, unsweetened dairy) from those with unhealthy profiles (e.g., those high in fat, sugar, and/or salt, but low in essential nutrients), and to understand their determinants [8].

The strong influence of parental eating behaviour and feeding practices on children’s diet is well established [6,13]. However, there is less understanding of parental influences, specifically on children’s snacking behaviours [14,15], i.e., energy consumption between meals, particularly in relation to how different healthy or unhealthy snacking habits, might contribute to children’s risk of obesity [16]. A recent systematic review of 47 studies on parenting practices that included information on children’s snacking found that 39 of these studies concerned parenting practices not specifically related to snacking [17]. Furthermore, the most consistent significant associations were that parental restriction of food was related to higher levels of child snacking, which suggests reactive rather than proactive parenting [17]. This reactive parenting appears to be a common response [18], with such parental food restriction also being positively associated with child obesity [19]. By contrast, there is a paucity of evidence for beneficial effects of proactive parenting, such as modelling, on children’s snacking *per se*, although the shared family environment is known to explain most of the variance in snack preferences in preschool children [17,20].

Baseline data from the ToyBox-study (www.toybox-study.eu), a six-country kindergarten-based, family-involved intervention to improve energy-balance related behaviours in preschool children [21], provided the opportunity to examine cross-sectional relationships between parental feeding practices and their children’s snacking, in a large and diverse international, yet thoroughly observed, sample. ToyBox-study surveyed parents from six European countries in detail about their attitudes to snacking, their practices in feeding their children, and their own and their children’s frequencies of healthy and unhealthy snacking. At the time, these data were collected, there had been little research into relations between parental practices and young children’s snacking behaviours, with the exception of the development of the Toddler Snack Food Feeding Questionnaire [22]. Moreover, that study only examined associations between parental practices and unhealthy snacking, and found little association with child obesity. More recently, Davison et al. [15] conducted a qualitative study while using semi-structured interviews of caregivers’ snack feeding practices for preschool children. They proposed four dimensions: autonomy support (praise/ support/modelling of healthy snacking/child-centered provision); structure (planning/ routine/availability/monitoring); coercive control (snacks to manage behaviour/unilateral decisions/restriction/pressure); and, permissiveness (no rules/disinterest/emotion-based feeding). By comparison, a quantitative cluster analysis of a 21-item ‘Comprehensive Snack Parenting Questionnaire’ [23] reported four-dimensional clusters of parental practices: “high covert control and rewarding”, “low covert control and non-rewarding”, “high involvement and supportive”, and “low involvement and indulgent”. Parents practising “high involvement and supportive” feeding had children who tended to eat energy-dense snacks least often.

The ToyBox-study questionnaires completed by parents included various items that were related to snacking practices, and in relation to parental nutritional knowledge. Our interest was primarily in examining supra-country patterns for both healthy and unhealthy snacking using hierarchical modelling to control for country differences, although some differences in unhealthy snacking between countries have been reported for the ToyBox-study [24]. Thus, as in the above studies, the present study focused on examining the dimensional structure of responses (grouping of item response variation) on items that were related to parental feeding practices, as being behaviourally and psychometrically more meaningful than examining associations with individual questionnaire items. Therefore, we applied multilevel modelling to determine the independent influences of these parents’ feeding practices, their own snacking, and the parents’ nutritional knowledge in relation to snacks, on both healthy and unhealthy snacking by their children.

## 2. Materials and Methods

### 2.1. Study Design

The Toybox-study was an intervention with a cluster randomized design, having two phases of data collection, at the baseline and at post-intervention (2012–2013). A detailed description of the ToyBox-study design is provided elsewhere [25]. The present study considers the baseline data only. The missing data were not replaced. The ToyBox-study adhered to the Declaration of Helsinki and the conventions of the Council of Europe on human rights and biomedicine. All the countries (Belgium, Bulgaria, Germany, Greece, Poland, and Spain) obtained ethical clearance from the relevant ethical committees and local authorities; all the parents/caregivers provided a signed consent form before being enrolled in the study.

### 2.2. Participants

Parents or primary caregivers (97% mothers) and their children attending kindergarten while aged 3.5–5.5 years old were recruited to the ToyBox-study, a European Commission-funded large-scale kindergarten-based, family-involved intervention in preschool children, and their families from six European countries: Belgium, Bulgaria, Germany, Greece, Poland, and Spain.

The study sample was composed of preschool children and their families, who were approached via kindergartens that were recruited from three socioeconomic levels of municipalities within each country. All the data presented in the present study were obtained between May and June 2012, while using standard methods and equipment that were applied by trained researchers. Initially, 10,632 parents/caregivers of preschoolers from six European countries provided written informed consent to participate in the ToyBox cross-sectional study. Of these, 8117 parents/caregivers (76.3%) filled in the CORE Questionnaire, and 7244 parents/caregivers (68.1%) filled in the Food Frequency Questionnaire [24]. Demographic information is not available on those parents who failed to participate in questionnaire completion.

Family, sociodemographic, and behavioural data were self-reported by parents and caregivers via the CORE-questionnaire (CORE-Q), including their years of education, weight and height, and their child’s sex, and details regarding parental feeding practices and attitudes, which were derived from extensive focus group research and shown to be reliable [26]. Parents also completed a highly structured food frequency questionnaire concerning their child’s diet (Child FFQ); this has been validated in a subsample from the ToyBox-study, with moderate to good validity, including for snack foods, such as biscuits [27]. As this study specifically concerns the influence of parental feeding practices, to improve the validity of any associations between the feeding practices and/or obesity of the primary carer and that of the child, we only analysed parent/guardian-child dyads, where the primary carer who completed the questionnaires was also the person who usually cooked for the child and fed the child, as determined by the responses to relevant questions on the CORE-Q. The questionnaires can be found online (www.toybox-study.eu) and detailed information on the development, test-retest reliability, and validity has been described elsewhere [26,28,29].

### 2.3. Predictor Variables

#### 2.3.1. Derivation of Scales for Parental Attitudes and Practices Regarding Snacking

At the time this study was designed, behavioural models specifically characterising parental feeding practices in relation to child snacking were not available, although three questionnaires have been recently developed in this area [22,23,30]. Nevertheless, as parental practices were of interest in the ToyBox-Study, the CORE-Q contained relevant questions. Although the large multinational sample and intervention design did not allow for a standard psychometric validation, we were interested in examining the possible dimensional structure of the set of questions on this topic, and so applied psychometric analyses to determine this. Thus, suitable items were selected from the CORE-Q based on representing specific beliefs, attitudes, and practices of the parents in regard to their child’s snacking behaviour. This section of the questionnaire was headed “Please read the following statements and tick the boxes most appropriate to your situation for morning, afternoon and evening snacks (responses on a five-point Likert scale, from 1 = “strongly disagree” to 5 = “strongly agree”). An initial item analysis was carried out, and after examination of the correlation matrix, some items were excluded as being too similar to others: the remaining 12 items had acceptable distributions, with skewness ranging from 0.05 to −1.06, means ranging from 2.12 to 4.18 (on a 1–5 scale), and the smallest SD = 0.78. Therefore, Principal Components Analysis (PCA) was used to assess these items for potential contribution to unique scales with Promax non-orthogonal rotation. The pattern matrix (Table 1) suggested four factor components (2–4 items per factor with loadings above 0.40). These are labelled as follows: component 1 (3 items) = ‘Healthy snack provision’; component 2 (2 items) = ‘Permissive healthy snacking’; component 3 (3 items) = ‘Unhealthy snacks child responsive’; and, component 4 (4 items) = ‘Permissive unhealthy snacking’. Thus, the scale scores were generated from the means of the respective items, having reverse-scored items 9 and 12 (Table 1) for the component 4 scale. These four derived variables were normally distributed. For scales consisting of 2–4 items only, within-scale reliability is likely to be limited [31], but the interest here is in dimensionality derived from the PCA [32]; nevertheless, Guttman’s λ_2_ values [33] were computed, as follows: healthy snack provision λ_2_ = 0.60; permissive healthy snacking λ_2_ = 0.73; unhealthy snacks child responsive λ_2_ = 0.51; and, permissive unhealthy snacking λ_2_ = 0.49.

#### 2.3.2. Parent/Carer Nutritional Knowledge for Snacks

Questions that were related to parental nutritional knowledge were embedded in the CORE-Q. We used these to develop a measure of nutritional knowledge in relation to snack foods, by establishing a scoring system that was based on expert consensus. Our scoring system incorporated recommendations for both healthy and unhealthy snack foods, and quantified disparity from those recommendations, which allowed for a more nuanced and varied scale scoring than previously derived from this questionnaire [34]: moreover, our method is similar to one that has previously been shown to result in more general nutritional knowledge scores being predictive of children’s eating behaviour [35]. A summary measure of parents’ understanding of recommended healthy snacking patterns was derived from responses to a question “What do you think is an acceptable consumption of the following food items for 4–6 year-old children?” for each of 10 possible food categories. There were eight possible responses: never; on certain occasions i.e., birthdays; 1 or less times per week; 2–4 times per week; 5–6 times per week; 1–2 times per day; 3–4 times per day; and, 5 or more times per day. The food categories were: sweets/candies/chocolate; biscuits/cookies/cakes/muffins; crisps and other similar salty snacks; fruit and vegetables; pizza, cheese pies/meat pies; milk (plain); yogurt (plain); milk (flavoured); yogurt (flavoured); and, cheese.

The present study interpreted recommendations for snack food consumption based upon the dietary recommendations of the Belgian Health Council, the World Health Organization, and the advice for school food standards for England [36], combined with data on habitual dietary intake in the Belgian population [37] since food-based dietary guidelines (FBDG) for preschool children only exist on a national basis. These FBDG are very similar to dietary guidelines in other countries [38], which makes these guidelines applicable for a European population of preschoolers [39].

On this basis, expert consensus was achieved (Toybox-study Group) on what frequencies of consumption would be recommended for each of these categories, for four to six year-old children, as shown in Table 2, with the exception of cheese for which no consensus was reached. For each category, recommendations encompassed two or three possible frequency responses. Subsequently, responses were recoded to indicate the closeness to the recommendations by giving a maximum score to responses equal to the recommendations, and then one point was subtracted for each frequency level by which the responses differed from recommendations. The maxima were set at either six or seven, depending on whether recommended frequencies spanned two or three levels, so that the minimum possible score for any category was one. For example, if a caregiver indicated that they believed that salty snacks were recommended to be eaten ‘1 or less times per week’, i.e., one level above the nearest recommended frequency, they would be scored as 6 − 1 = 5 for that response.

These individual snack category knowledge scores were summed to provide an overall snack nutritional knowledge score. As this score was negatively skewed, the variable was reversed, then natural log transformed, and then unreversed to maintain the original score direction. This final transformed variable was normally distributed.

#### 2.3.3. Parent/Carer Snack Modelling Behaviour

From the CORE-Q, the principal parental modelling variables were derived from parents’ reports of frequency of consuming various snack foods, from a question asking “How often do you consume the following items as a snack (in between your main meals)?”. The foods listed were: Nuts/peanuts; Cakes/muffins; Wholemeal Bread; Biscuits/cookies; Crisps and other similar salty snacks; Crackers, breadsticks; Chocolate; Sweets/candies; Cheese; Cheese pies/meat pies; Yogurt/fresh cheeses; Pizza; Fresh fruits; and, Vegetables.

Frequency categories were: never; 1 or less times per week; 2–4 times per week; 5–6 times per week; 1–2 times per day; 3–4 times per day; and, 5 or more times per day. The frequencies were recoded to times per day using mid-point frequencies as follows: 0; 0.143; 0.429; 0.786; 1.5; 3.5; and, 5.

These were used to form a total frequency per day of consuming healthy or unhealthy snack foods, by summing two groups of snack food categories, as follows: ‘healthy snacks’: nuts, wholemeal bread, yogurt, fruits, vegetables; ‘unhealthy snacks’: cakes, biscuits, salty snacks, chocolate, sweets, pies, and pizza. These variables were positively skewed and so were transformed by natural logarithm. The transformed variables were normally distributed.

A general parent snacking frequency variable was derived from questions asking “How often do you usually have something to eat as snack between the meals during weekdays, for morning, afternoon and evening snack”, with frequency responses ranging from “never” (scored 0), then “On 1 day” up to “on 5 days”. The same questions were asked about weekends but responses included “never”, “On 1 day” and “On 2 days”. The responses were summed to provide a score of overall frequencies of snacking per week. This variable was normally distributed (range 0 to 21, which is the full possible range).

### 2.4. Anthropometry

Two consecutive measurements of children’s weight to the nearest 100 g while using electronic scales (types SECA 861 and SECA 813; Seca, Hamburg, Germany) and height to the nearest 0.1 cm using a portable stadiometer (types SECA 225 and SECA 214; Seca) were taken. For anthropometric measurements, the children were measured in light clothing without shoes, and were asked to stand still in an erect position. Body mass index (BMI; weight, kg/(height, m)^2^) z-scores (zBMI) were calculated with use of the LMS method and children were categorized as normal weight or overweight/obese [40]. Parental age, height, and weight were obtained by self-report on the CORE-Q. Parental BMI was positively skewed and so was transformed to a non-skewed distribution by natural logarithm prior to parametric statistical analyses.

Resting energy expenditure (REE) for the children (3–10 years old) was calculated while using the Schofield equation, including both height and weight, as recommended for such populations [41]. This enabled energy needs to be controlled for when, for example, relating child zBMI to snack intake variables.

### 2.5. Dependent Variables

#### 2.5.1. Child snack Intakes from Child Food Frequency Questionnaire

Two variables were derived from the Child FFQ: first, daily intakes were estimated from the product of the frequency of consumption per day and the standard portion size for that food, as specified on the questionnaire. The intakes of likely snack foods were then grouped into ‘healthy’ and ‘unhealthy’ lists, as follows: Healthy snack foods: Plain yogurt; cheese; fresh fruit; raw vegetables; unsweetened cereals; wholemeal bread and similar bakery products. Unhealthy snack foods: chocolate; cakes; biscuits; pastries; sweet spreads; salty snacks; and, sugar-based desserts.

Intakes within each group were summed to provide child healthy and unhealthy snack intake variables. The child unhealthy snack intake variable was positively skewed and so was transformed by a natural logarithm. The transformed variable was normally distributed.

#### 2.5.2. Child Snacking Tendencies

An overall estimate of frequency of child snacking behaviour was derived from the question “How often does your child eat something in between meals?”, with the response frequencies ranging from “never or less than once a month” to “every day”. The responses were recoded into child snacking frequency per week. However, this variable was somewhat bipolar distributed; therefore, the variable was recoded to form a dichotomous categorical variable, where those children reported to snack either 5–6 days a week or every day were classified as ‘snacking most days’ (60.6%), and the remaining children were classified as ‘not snacking most days’ (36.9%; 2.5% missing).

### 2.6. Alpha Level Adjustment

Alpha level for statistical significance was set at 0.01 due to the large sample size and large number of tests examined.

### 2.7. Data Analysis

Simple correlations were tested with Pearson’s r and partial correlations (r_p_), with variables being transformed to normalise if needed. For adjusted models, multilevel modelling was employed to predict child snack intake, using nlme package [42] in R [43], as this acknowledges the hierarchical structure of the data and it is robust against missing values [44]. Two hierarchical models were built, modelling healthy and unhealthy child snack intake. For both models, a two-level hierarchical structure was utilized (i.e., country/child). To be precise, each child with information on child anthropometry, parental attitudes, knowledge, modelling behaviour, and educational status as predictors was nested within the country information. Allowing for intercepts to vary across countries significantly improved the model fit for predicting child healthy snack intake (χ^2^(1) = 275.43, *p* < 0.001), and child unhealthy snack intake (χ^2^(1) = 60.20, *p* < 0.001), thus justifying the use of multilevel analyses.

For each analysis, adding predictor variables one by one and comparing the current model with the previous one, to develop a final model, created multiple models with fixed effects. Chi-square (χ^2^) test of change in log-likelihood with full maximum-likelihood estimation (ML) was used to compare a model fit of different models [45]. The list of relevant predictor variables for each model was decided prior to the analyses and no predictors were excluded from the model due to lack of significance in order to evaluate whether and how much each hypothesised variable predicted the relevant outcome. Once fixed effects were modelled, the existence of random effects was also evaluated.

## 3. Results

### 3.1. Participants

From the original sample of 7076 parent-child dyads, 5185 were available for analyses, which included parents reporting involvement in cooking for and feeding their child. Of this sample, 97% of participating parent/caregivers were mothers, 2.4% were fathers, the remaining 0.6% being step-parents or classified as other; the parent/caregiver is referred to here as ‘parent’. There were 2685 (51.8%) boys and 2500 (48.2%) girls. Table 3 provides descriptive data for the parents and children.

### 3.2. Differences in Child zBMI and Snack Consumption by Country

Child zBMI significantly varied across the six countries (Table 4: one-way ANOVA, country effect, F(5, 4994) = 18.1, *p* < 0.001), with the most marked difference being that zBMI for Greece was higher than all other countries.

For frequency of snacks per week, Belgium showed the highest, whereas Spain showed the lowest frequency when compared to the other countries (Table 4: one-way ANOVA, country effect, F(5, 5052) = 271.2, *p* < 0.001; REE was not a significant covariate for snacking frequency in ANCOVA).

Healthy and unhealthy snack intakes also varied by country but contrasted with differences in snacking frequency. For healthy snack intake, Belgium and Spain showed the lowest intakes, and Bulgaria the highest (Table 4: adjusted for child REE, ANCOVA country effect, F(5, 4965) = 63.9, *p* < 0.001; REE covariate, F(1, 4965) = 33.4, *p* < 0.001). For unhealthy snack intake, Belgium was notably higher than all other countries (ANCOVA on Ln-transformed data adjusted for REE, country effect, F(5, 4957) = 20.9, *p* < 0.001; REE covariate, F(1, 4965) = 4.01, *p* < 0.05). We also report the ratio of healthy to unhealthy snack intake, since this provides an indicator of the healthiness of snacking behaviour, independent of overall snack intake: this again varied across countries, with Belgium having the least healthy profile (lowest ratio) as compared to other countries, although not significantly different from the next lowest, Spain (Table 4: one-way ANOVA, country effect, F(5, 5091) = 11.2, *p* < 0.001).

### 3.3. Is Parental Snacking Behaviour Associated with Child Healthy and Unhealthy Snacking?

The overall estimate of parent’s weekly snacking frequency was unrelated to child healthy snack intake, but it was associated with greater child unhealthy snack intake (Table 5). Parents’ healthy snacking frequency was associated with greater child healthy, but not unhealthy, snack intake. Conversely, parents’ unhealthy snacking frequency was associated with greater unhealthy, but not healthy, child snack intake.

Furthermore, children who snacked on most days of the week had parents who snacked more often (*n* = 3193, mean [SD] = 11.04 [5.52] times per week) than did children who did not snack on most days of the week (*n* = 1798, mean [SD] = 8.37 [5.29] times per week), t(3861.3) = 16.89 *p* < 0.001; adjusted for unequal variances, Levene’s F = 6.80, *p* = 0.009).

### 3.4. Are Parental Attitudes and Practices Concerning Snacking Associated with Child Healthy and Unhealthy Snack Intakes?

Providing healthy snacks, and being permissive about allowing healthy snacking were both associated with greater child healthy snack intake, but not with child unhealthy snack intake. Conversely, allowing for unhealthy snacking was strongly associated with greater child unhealthy snack intake, but negatively related to healthy snack intake (Table 5). Similarly, responding to child demands for unhealthy snacks was associated with higher unhealthy snack consumption and less healthy snack consumption by their children.

Greater parent/caregiver understanding of recommendations for snack food consumption for young children was associated with more child healthy snack intake and less child unhealthy snack intake. Moreover, parents with more years of education also had greater knowledge regarding snack recommendations (Pearson’s r(5030) = 0.126, *p* < 0.001).

These associations, although cross-sectional, suggest that parental snacking behaviour and practices may be influencing both child healthy and unhealthy snack consumption.

### 3.5. Associations between Snacking Behaviours and Child Obesity

In zero-order correlations, child zBMI was positively associated with children’s reported intake of healthy snacks (r(4972) = 0.051, *p* < 0.001), but unrelated to unhealthy snack intake (r(4964) = 0.007, *p* = 0.612). More parental years of education was associated with higher child healthy snack intake (r(5050) = 0.081, *p* < 0.001) and lower unhealthy snack intake (r(5042) = −0.042, *p* = 0.003), and with lower child zBMI (r(4895) = −0.071, *p* < 0.001).

However, because zBMI is strongly related to REE (r(5000) = 0.667, *p* < 0.001), which in turn could drive snack intake, partial correlations were used to control for any influence of REE. zBMI was no longer associated with either healthy (r_p_(4953) = −0.005, *p* = 0.699) or unhealthy (r_p_(4953) = 0.000, *p* = 0.994) snack intake with these adjusted partial correlations.

In line with this lack of relationship between zBMI and child snacking, only one significant relationship for snacking attitudes and practices of parents was seen with child zBMI among these partial correlations: children with higher zBMI had parents who tended to report being less permissive regarding healthy snacking by their child (r_p_(4819) = −0.060, *p* < 0.001). Knowledge of snack recommendations was weakly positively related to zBMI in these adjusted analyses (r_p_ = 0.029, *p* = 0.041), although not significantly at *p* < 0.01. Neither permissiveness nor responsiveness for unhealthy snacking, nor provisioning for healthy snacking, were related to zBMI in these partial correlations (respectively, r_p_ = 0.011, 0.001, 0.008).

### 3.6. Hierarchical Multilevel Modelling of Predictors of Child Snack Intake

Hierarchical linear regression, or multilevel modelling is the more powerful method for testing for adjusted associations in these hierarchical data. These models adjust for any potentially significant variance due to differences between kindergartens or countries; in this case, the best model used two levels, child nested in country. Table 6 and Table 7 present these regression results for predictors of child healthy and unhealthy snack intake, respectively.

In addition to REE, which was a strong predictor, child healthy snack intake was independently associated with having parents who had more years of education, had better knowledge of snack recommendations, ate more healthy snacks, provided more healthy snacks to their children, and were more restrictive of unhealthy snack consumption (Table 6). Child zBMI was unrelated to healthy snack intake.

In contrast, for child unhealthy snack intake, REE was relatively weakly positively related, implying mainly non-regulatory (e.g., hedonic) drivers to eat such snacks (Table 7). Children who ate more unhealthy snacks had parent/carers who ate more unhealthy snacks, were less restrictive of their children eating such snacks, had fewer years of education, and were less knowledgeable about snack recommendations. Child unhealthy snack intake was also unrelated to parents’ healthy snacking, implying a specific association to the class of snack.

## 4. Discussion

This study has applied some novel behavioural measures of parental attitudes, as well as their own snacking behaviour, to both healthy and unhealthy child snacking behaviour, in a cross-sectional analysis of a large and well-measured sample of 3.5–5.5 year-old children. The findings contribute to the limited existing literature on this topic [30], and they provide some useful and novel understandings of the differential influences of parental behaviour on healthy and unhealthy snacking by their children, and implications for obesity risk in young children.

There was clear evidence that parental habitual snacking was associated with, and likely influenced, their child’s snacking: thus, for the specific parental snack frequency variables, parental healthy and unhealthy snack intake predicted respective child healthy and unhealthy snack intake, but not vice-versa. Furthermore, children who snacked on most days had parents who reported snacking more frequently than did the parents of children who did not snack on most days. These associations could reflect the child acquiring snacking habits through parental modelling of snacking, although other mechanisms could be involved, including exposure and opportunity via snack availability in the home. Our data do not allow for us to distinguish these possibilities for certain, although the parent/carer was specifically the main feeder of their child here, parent/carer snack intake was predictive of child snack intake independently of a measure of snack provision, and parental modelling has been shown to promote both snacking [46,47] and fruit and vegetable consumption [35,48,49].

The parental feeding strategies for young children are known to result in differential effects on the healthiness of their children’s diets [50,51]. Here, being permissive about their child’s snacking promoted only unhealthy snack intake, whereas children’s healthy snack intake was higher in parents who were specifically less permissive of, or less responsive to, unhealthy snacking. It is possible that the more hedonically attractive, and energy-dense, nature of unhealthy snacks makes them more amenable to such parental strategies or simple modelling effects than might be the case for healthier snacks, at least in young children [52,53]. A key variable that was not directly assessed here might be expressed preference as opposed to just intake, as in another study maternal food preferences were an important predictor of children’s intake of healthy foods but not unhealthy foods [53]. Nevertheless, in this study, healthy snack provision did appear to promote specifically healthy snack intake, independent of parental intake, nutritional knowledge, or education. It should also be noted that these results are independent of country differences, as this level was controlled for in the multilevel modelling. Nevertheless, we replicated the earlier report from ToyBox-study data that Belgium children appear to most frequently snack [24], and extend this to show that their snacking profile was the least healthy of the six countries. Presumably, the country differences in snacking reflect a combination of cultural, economic, environmental, and food availability influences.

To summarise, beyond potential modelling effects from eating snacks (to the extent to which this happened in the child’s presence), our findings from the measures representing parental snack feeding practices were also significantly associated with the children’s healthy and unhealthy snack consumption. On the positive side, parents’ practices of making healthy snacks available to their children was associated with greater child healthy snack intake, but it did not relate to unhealthy snack intake. Conversely, parental practices that are related to unhealthy snacking, i.e., being permissive about unhealthy snacking including making them available, and being responsive or acceding to child demands for unhealthy snacks, was associated with the greater consumption of unhealthy snacks by children, but also less intake of healthy snacks. This suggests that when unhealthy snacking is encouraged, their intake displaces healthy snacks from children’s diet, although the reverse might not be the case.

In addition to these parental practices regarding their children’s snacking, parents having greater nutritional knowledge of snack food recommendations had children who ate more healthy snacks and fewer unhealthy snacks. This measure was a strong predictor of child healthy vs. unhealthy snacking, independent of education level: thus, this finding supports the use of nutritional education for parents as an aspect of interventions addressing children’s diets and obesity risk [34]. The weak tendency for higher child zBMI to be associated with greater parental nutritional knowledge was an unexpected finding: if not a chance result, it could perhaps indicate some reverse causality, i.e., greater interest in dietary recommendations among parents with more overweight children.

The associations between parental snacking practices and their children’s snacking shows that such parenting techniques are significant influences on healthy and unhealthy snacking in these young children: thus, practitioners should educate parents regarding the importance of their own behaviours, as well as instilling knowledge of snack food group recommendations, whilst being aware that less educated parents are particularly at risk of unhealthy parental practices [54,55]. Furthermore, such advice is supported by evidence that interventions, such as the ToyBox-study, can improve relevant parental snacking practices and rule-setting, as well as nutritional knowledge of parents [34]. However, there was little support here for associations between child snacking and child obesity, which is in agreement with a previous analysis of associations between unhealthy snacking and baseline BMI in these children, although soft drink consumption, which was not examined here, was found to be positively related to BMI [56]. One reason might be that this age range is approaching the age (5–6 years old) at which BMI typically reaches a minimum (‘adiposity rebound’), thus limiting the variance in BMI, before rising again through to adulthood. The limited associations between either child snacking or parental snacking practices and child obesity are also consistent with other studies of parental and child snacking behaviours and obesity in similar aged young children [22,30]. Still, children starting the adiposity rebound at a younger age are much more likely to be obese in adulthood [57], so preschool child overweight or obesity is still a concern; furthermore, child obesity dramatically increases during early school years, and unhealthy energy-dense snacking is likely to be a contributory factor [12]. Moreover, we did find evidence that parents who were less permissive in encouraging healthy snacking had children with higher zBMI; this might indicate that healthy snacks, which are generally lower in energy density than unhealthy snacks, could help displace unhealthy snacks and so reduce the children’s overall energy intake.

With this in mind, particularly from a research perspective, an innovation in this study was to include REE as a predictor alongside BMI, in an attempt to separate the impact that greater energy needs *per se* might have on snack intake from other behavioural characteristics that may be inherently linked to being a heavier child. For instance, snacking, especially as defined by eating energy-dense food between meals, has been considered to be an expression of ‘eating in the absence of hunger’, which has been associated with an increased adiposity in young children [8,58], perhaps due to weaker appetite regulation in more overweight children [59], which in turn could result from parental use of controlling feeding practices [60]. However, the inclusion of REE in the regression analyses resulted in REE, but not zBMI being a significant predictor of both healthy and unhealthy snack intake in our fully adjusted multilevel models. It should also be noted that, despite zBMI being greater in Greece than the other intervention countries, as was previously observed [61], our hierarchical analyses controlled for such country-level variation. Furthermore, although sex differences in the associations between fruit and vegetable intake and child obesity have been reported for this ToyBox-Study cohort (albeit a smaller sample) [56], we did not find an effect of sex on healthy or unhealthy child snack intake in our multilevel models, including REE. Therefore, investigators should consider controlling for differences in physiological energy requirements that vary with body mass, age, and sex when examining relationships between children’s snack intake, obesity and potential behavioural risk factors; then, for example, any associations between snacking-related behaviours and child obesity would not be confounded by greater energy needs in more overweight or at least heavier, older, or faster-growing children.

This study is limited by the self-report nature of the behavioural variables, and the development of new measures without full psychometric validation. A further limitation of our observations is that the effect sizes were generally moderate to very small, and the associations cross-sectional: thus, it must be acknowledged that other factors, some of which may covary with snacking, which have not been addressed here, could play more important roles in linking parental and child behaviour to child obesity risk [62,63]. It is also possible that unhealthy snacking has not yet had time to influence the development of obesity in these young children; however, we only examined cross-sectional relationships at baseline in order to benefit from the largest sample size.

One factor not measured here that could disrupt simple associations between overweight and obesity, healthy or unhealthy snacking, parental practices, and indeed fruit and vegetable intake, is the children’s fussy eating tendencies, since fussy eaters will tend to avoid snacking on healthy foods, yet also tend to be thinner [64,65]. In fact, parents often struggle to find successful strategies for promoting healthy eating in picky/fussy children [66]. Moreover, it is likely that associations between energy-dense snack consumption and obesity will increase with age of the child [56,58].

## 5. Conclusions

Child obesity was unrelated to their (healthy or unhealthy) snack intake, in adjusted models that included energy requirements, parental education, and feeding practices, perhaps reflecting the young age of the children and the greater importance of parental influences. Clear independent predictors of both healthy and unhealthy snacking included knowledge of dietary recommendations for snack foods (negative for unhealthy snacking), parent/carer education, and parent/carer snack intake. Unhealthy snacking can lead to dietary habits that are harmful to health, irrespective of obesity, and these findings could help to support interventions for improving children’s nutritional status, as well as the dissemination of healthy dietary practices to parents of young children.

## Figures and Tables

**Table 1:** Pattern matrix from PCA with Promax rotation^a^ of parent/carer attitudes and practices from CORE-questionnaire (CORE-Q) item responses related to child snacking.

CORE-Q Items	Component			
	1	2	3	4
1. I make dairy snacks regularly available for my child	**0.812**			
2. I make cereals/bread snacks regularly available for my child	**0.727**	0.136	0.141	
3. I make fruit or vegetables snacks regularly available for my child	**0.625**		−0.276	
4. My child is allowed to eat dairy or cereals/bread as snacks without asking	0.141	**0.849**		
5. My child is allowed to eat fruits or vegetables as snacks without asking		**0.838**		
6. If I prohibit my child to eat a sweet or salty snack, I find it difficult to stick to my rules if he/she starts nagging	−0.119	0.106	**0.733**	
7. I find it difficult to restrain myself from eating sweet or salty snacks because of the presence of my child	0.168	−0.169	**0.686**	
8. I give sweet or salty snacks to my child as a reward or to comfort him/her	−0.126		**0.663**	
9. My child is allowed to eat sweet or salty snacks only at certain occasions i.e., birthdays	0.228		0.177	**−0.726**
10. I make sweet or salty snacks regularly available for my child	0.145		0.153	**0.707**
11. I think eating sweet or salty snacks is not bad for my child	0.135	−0.276		**0.603**
12. My child is not allowed to snack while watching TV		−0.324		**−0.454**

Note: Extraction Method: PCA. ^a^ Rotation Method: Promax with Kaiser Normalization. Rotation converged in 6 iterations. Item loadings in bold indicate those used for that scale.

**Table 2:** Description of recommendations for frequency consumption of different snack food categories for preschool children.

Snack Food Category	Recommended Frequency of Consumption
sweets/candies/chocolate	Never to 1 or less times per week
biscuits/cookies/cakes/muffins	Never to ‘on certain occasions’
crisps and other similar salty snacks	Never to ‘on certain occasions’
fruit and vegetables	3–4 times to 5 or more times per day
pizza, cheese pies/meat pies	‘On certain occasions’ to ‘1 or less times per week’
milk (plain)	1–2 to 3–4 times per day
Yogurt (plain)	1–2 to 3–4 times per day
milk (flavoured)	Never to 1 or less times per week
yogurt (flavoured)	Never to 1 or less times per week

Note: Based on national guidelines and expert consensus from the ToyBox-study Group (see text for references).

**Table 3:** Descriptive statistics for participant characteristics.

	N	Mean	SD	Min.	Max.
Child Age (years)	5183	4.75	0.43	3.50	5.49
Parent/Carer Age (years)	5158	35.60	4.82	21	66
Parent BMI	5080	23.49	4.14	15.23	60.24
Child zBMI	5000	0.22	1.03	−5.87	4.01
Child healthy snack intake (g/d)	5156	256.1	139.7	0.0	842.9
Child unhealthy snack intake (g/d)	5149	57.3	42.4	0.0	374.6
Child resting energy expenditure (kcal/d)	5000	883.65	61.66	701.06	1276.50

**Table 4:** Differences by country in child zBMI and measures of healthy and unhealthy snack consumption, and the ratio of healthy to unhealthy snack intake. Data are expressed as means (95% CIs).

Country	N Range ^1^	Child zBMI	Snacks Per Week	Healthy Snack Intake ^2^ (g/d)	Unhealthy Snack Intake ^2^ (g/d)	Healthy: Unhealthy Snack Ratio ^3^
Belgium	692–700	0.22 ^a^ (0.15–0.29)	6.11 ^a^ (5.99–6.23)	219.5 ^a^ (209.3–229.6)	67.1 ^a^ (63.9–70.3)	4.24 ^a^ (3.97–4.51)
Bulgaria	580–606	0.24 ^a^ (0.16–0.32)	5.13 ^bc^ (4.95–5.30)	328.9 ^b^ (318.0–339.8)	64.2 ^b^ (60.7–67.6)	10.26 ^b^ (8.56–11.96)
Germany	871–948	0.06 ^b^ (−0.01–0.12)	5.43 ^b^ (5.30–5.56)	279.7 ^c^ (270.7–288.7)	50.4 ^bc^ (47.6–53.3)	8.38 ^bc^ (7.43–9.33)
Greece	1258–1306	0.43 ^c^ (0.37–0.49)	5.43 ^b^ (5.31–5.55)	248.3 ^d^ (240.8–255.8)	56.9 ^c^ (54.5–59.3)	8.60 ^bc^ (7.60–9.59)
Poland	1055–1065	0.10 ^ab^ (0.04–0.16)	4.81 ^c^ (4.69–4.95)	248.2 ^d^ (240.1–256.2)	55.4 ^c^ (52.9–58.0)	8.64 ^bc^ (7.47–9.81)
Spain	517–535	0.21 ^ab^ (0.12–0.30)	2.00 ^d^ (1.79–2.20)	209.6 ^a^ (198.0–221.2)	53.8 ^bc^ (50.2–57.5)	6.58 ^ac^ (5.69–7.48)

^1^ Sample sizes varied slightly for complete data for each measure within countries. ^2^ Estimated marginal means adjusted for child resting energy expenditure as a significant covariate. For unhealthy snack intake, analyses were performed on Ln-transformed data. ^3^ Healthy snack intake divided by unhealthy snack intake, excluding zero values for either variable; a higher number represents a healthier snacking profile. ^abcd^ Differing superscript letters within each column indicate significant differences between countries (Bonferroni multiple comparisons).

**Table 5:** Correlations (Pearson’s r) between parent snacking practices and child snacking behaviour.

Parental Predictor Variables	Child Healthy Snack Intake ^a^	Child Unhealthy Snack Intake ^b^
Parent healthy snacks/day	0.204 **	−0.014
Parent unhealthy snacks/day	−0.009	0.255 **
Parent snacks/week	0.008	0.121 **
Healthy snack provision	0.115 **	0.018
Permissive healthy snacking	0.075 **	0.006
Unhealthy snacks child responsive	−0.150 *	0.157 **
Permissive unhealthy snacking	−0.136 **	0.249 **
Knowledge of snack recommendations	0.166 **	−0.210 **

* *p* < 0.01, ** *p* < 0.001 for significant Pearson’s r correlation (2-tailed). Significance also indicated in bold. ^a^ N ranges from 5088 to 5122; ^b^ N ranges from 5081 to 5115.

**Table 6:** Hierarchical linear regression statistics for predictors of child healthy snack intake.

Predictor	B (SE)	t(4700)	Predictive *p* Value
Child zBMI	1.43 (2.48)	0.58	0.565
**Resting energy expenditure**	0.18 (0.04)	4.24	<0.0001
Parent unhealthy snack intake	3.30 (1.32)	2.50	0.013
**Parent healthy snack intake**	5.23 (0.59)	8.84	<0.0001
Parent snacking frequency	0.82 (0.37)	2.25	0.025
**Knowledge of recommendations**	32.99 (4.05)	8.14	<0.0001
**Unhealthy snack permissiveness**	−17.43 (3.17)	5.50	<0.0001
**Unhealthy snack responsiveness**	−16.21 (2.64)	6.15	<0.0001
Healthy snack permissiveness	−0.83 (1.97)	0.42	0.675
**Healthy snack provision**	28.50 (3.06)	9.33	<0.0001
**Parent/carer education level**	7.42 (1.70)	4.35	<0.0001

Note: The multilevel model accounts for variance at the levels of child (1) and country (2). Child zBMI was a significant fit in the model (Log Likelihood Ratio = 18.30, *p* < 0.0001) but not a significant multilevel predictor of child healthy snack intake. Sex and SES were not related to the dependent variable in this model. Strongest predictors shown in bold.

**Table 7:** Hierarchical linear regression statistics for predictors of child unhealthy snack intake.

Predictor	B (SE)	t(4693)	Predictive *p* Value
Child zBMI	−0.62 (0.77)	0.80	0.423
Resting energy expenditure	0.03 (0.02)	2.55	0.011
**Parent unhealthy snack intake**	4.90 (0.41)	11.83	<0.0001
Parent healthy snack intake	0.10 (0.18)	0.53	0.593
Parent snacking frequency	0.15 (0.11)	1.34	0.180
**Knowledge of recommendations**	−10.91 (1.26)	8.64	<0.0001
**Unhealthy snack permissiveness**	8.65 (0.98)	8.85	<0.0001
**Unhealthy snack responsiveness**	4.90 (0.82)	5.98	<0.0001
Healthy snack permissiveness	1.54 (0.61)	2.53	0.012
Healthy snack provision	0.89 (0.94)	0.95	0.341
**Parent/carer education level**	−1.64 (0.53)	3.10	0.002

Note: The multilevel model accounts for variance at the levels of child (1) and country (2). Sex and SES were not related to the dependent variable. Strongest predictors shown in bold.
